# Integrated optic nerve sheath diameter, glasgow coma scale and computed tomography based model for early prediction of clinically significant brain injury in head trauma: A prospective observational study

**DOI:** 10.6026/973206300223762

**Published:** 2026-06-30

**Authors:** Chandan K Dey, Merin John, Pallavi Shende, Varun Anand, Biraj Majumder, Sudarsan Behera, Ramaswamy N, Klein Dantis, Santosh K Rathia

**Affiliations:** 1 Department of Trauma & Emergency, All India Institute of Medical Sciences, Raipur, India; 2 Department of Emergency Medicine, Malankara Orthodox Syrian Church Medical College, Kerala, India; 3 Department of Orthopedics, All India Institute of Medical Sciences, Deoghar, India; 4 Department of Forensic Medicine and Toxicology, All India Institute of Medical Sciences, Raipur, India; 5 Department of CTVS, All India Institute of Medical Sciences, Bathinda, India

**Keywords:** Optic nerve sheath diameter (ONSD), glasgow coma scale (GCS), clinically significant brain injury (CSBI), point-of-care ultrasound, decision curve analysis, rotterdam score

## Abstract

Acute head injury requires rapid identification of clinically significant brain injury (CSBI), yet access to CT imaging is often limited, creating challenges in early triage. In this prospective study of 215 head trauma patients, optic nerve sheath diameter (ONSD) and Glasgow Coma Scale (GCS) were evaluated as predictors of CSBI. Patients with CSBI had significantly higher ONSD and lower GCS and both variables independently predicted injury severity. The combined ONSD + GCS model demonstrated better diagnostic performance, clinical utility and calibration than either parameter alone, with ONSD showing a dose-response relationship with CT severity. Thus, data shows the validating a practical bedside risk stratification approach that integrates ultrasound-derived ONSD with GCS to improve CSBI prediction, particularly in resource-limited settings.

## Background:

Head injury is a leading cause of death and disability, especially in low- and middle-income countries [1]. The Glasgow Coma Scale (GCS) is the standard bedside severity tool, but its accuracy is limited in intubated, sedated, or intoxicated patients [2]. Computed tomography (CT) is the gold standard for intracranial pathology, yet often unavailable in resource-limited settings. A rapid, non-invasive triage tool is needed to identify patients at high risk of clinically significant brain injury (CSBI). The optic nerve sheath is contiguous with the intracranial subarachnoid space; elevated intracranial pressure (ICP) distends the sheath and can be measured by ultrasound [3]. Optic nerve sheath diameter (ONSD) via point-of-care ultrasound is a promising non-invasive surrogate for raised ICP, with correlations to invasive ICP measurements and CT findings [4]. However, few studies have integrated ONSD with GCS into a predictive model for a composite CT-based outcome of CSBI. Moreover, the utility of ONSD for the most severe radiological phenotype (Rotterdam score ≥4) and the net clinical benefit of combining ONSD with GCS (via decision curve analysis) remain unexplored [5]. Therefore, it is of interest to evaluate the association of ONSD and GCS with CT-defined clinically significant brain injury and to assess the diagnostic utility of a combined ONSD-GCS model for early risk stratification in patients with head injury.

## Methods:

## Study design and setting:

A prospective observational study was conducted in the Department of Trauma and Emergency at a tertiary centre of Central India over 18 months. Ethical approval was obtained (IEC No: 2022/PG/ME/45) and the study followed STROBE and TRIPOD guidelines.

## Participants:

Consecutive patients aged >14 years with head injury and a history of loss of consciousness, vomiting, seizures, focal deficits, or ENT bleeding were enrolled. Exclusion criteria were suspected globe rupture or refusal of consent.

## Data collection:

GCS was assessed by trained emergency physicians on arrival. ONSD was measured 3 mm posterior to the globe using a high-frequency linear probe (7-13 MHz) on the closed eyelid with a SonoSite M-Turbo ultrasound machine; the average of both eyes was used. Non-contrast CT head was performed within 24 hours and reviewed by a blinded radiologist. The Rotterdam score (1-6, higher = worse prognosis) and Marshall grade (I-VI) were assigned. The presence of contusion, SAH, EDH, SDH and DAI was recorded dichotomously.

## Outcome definitions:

The primary outcome was clinically significant brain injury (CSBI), defined as a composite of Rotterdam score ≥3, Marshall grade III-VI, or the presence of SAH, SDH, or DAI on CT. Exploratory outcomes included Rotterdam score ≥4 (the most severe radiological phenotype), GCS-defined severe TBI (GCS 3-8, used descriptively) and ONSD trends across CT severity categories.

## Statistical analysis:

All analyses were performed in R version 4.3.2. Normality was assessed by Shapiro-Wilk test; non-parametric tests were used for skewed data. Continuous variables are presented as median (IQR) or mean±SD; categorical as frequencies (%). Baseline comparisons used Wilcoxon rank-sum (continuous) and chi-square (categorical) tests. Spearman's rank correlation assessed associations between ONSD, GCS, Marshall grade and Rotterdam score. Univariable logistic regression evaluated ONSD and GCS with CSBI. The primary multivariable model included ONSD_ avg, GCS.total and age group. Odds ratios (OR) with 95% CI are reported. Model fit was assessed by likelihood ratio test (with/without ONSD), AIC and Nagelkerke R^2^. ROC curves were constructed for GCS alone, ONSD alone and the combined model. AUC with DeLong 95% CI was calculated and compared. The optimal ONSD cut-off for CSBI was derived via Youden index; sensitivity, specificity, PPV and NPV are reported. Net reclassification improvement (NRI) at thresholds 0.3-0.6 and integrated discrimination improvement (IDI) were computed with 95% bootstrap CIs (1000 and 500 resamples, respectively). Bootstrap validation (1000 resamples) estimated optimism and corrected c-index. Calibration was assessed via calibration plot and mean absolute error. Decision curve analysis (DCA) plotted net benefit across thresholds 0-0.6 for the combined model, GCS alone, treat-all and treat-none. The Jonckheere-Terpstra test evaluated monotonic ONSD trend across Rotterdam and Marshall categories. Secondary multivariable logistic regression examined ONSD and GCS as predictors of Rotterdam ≥3 and Marshall ≥III.

## Exploratory analyses:

For Rotterdam ≥4 (subset of CSBI), an ROC curve for ONSD alone was constructed, AUC calculated and Youden cut-off derived (hypothesis-generating). Descriptive ONSD data (mean±SD) were provided for GCS-defined severe TBI (GCS 3-8) to illustrate dose-response, with no formal inference model to avoid circularity. A two-sided p<0.05 was considered significant.

## Results:

A total of 215 patients were included. The mean age was 36.4 ± 15.9 years (range 14-94) and 187 (87.0%) were male. Road traffic accidents were the most common mechanism (79%, n=169), followed by falls (9.3%, n=20), direct blows (4.2%, n=9) and assaults (3.3%, n=7) and other mechanisms (4.2%). The median GCS was 15 (IQR 14-15) and the median ONSD was 4.1 mm (IQR 3.9-4.8 mm). Overall, 48 patients (22.3%) met the criteria for CSBI. Table 1 summarises the baseline characteristics stratified by CSBI status. Patients with CSBI had significantly higher ONSD (median 4.8 mm vs. 4.1 mm, p<0.001) and lower GCS (median 13 vs. 15, p<0.001) compared to those without CSBI. There were no significant differences in age group (p=0.258) or gender (p=0.904) between the two groups. A significant negative correlation was observed between ONSD and GCS (Spearman's ρ = -0.348, p<0.001). A moderate positive correlation was noted between ONSD and Marshall Grade (ρ = 0.294, p<0.001), but ONSD did not correlate significantly with Rotterdam score (ρ = 0.122, p=0.074). In univariable analysis, ONSD (OR 2.66, 95% CI 1.73-4.18, p<0.001) and GCS (OR 0.70, 95% CI 0.60-0.80, p<0.001) were each strongly associated with CSBI. Gender and age group were not significant. In the multivariable model adjusting for GCS and age group, ONSD remained an independent predictor of CSBI (OR 1.74, 95% CI 1.04-2.92, p=0.033). GCS also remained independently associated (OR 0.73, 95% CI 0.62-0.85, p<0.001). Age group was not significant (p>0.05 for both comparisons). The full model results are shown in (Table 2). The ROC curves for GCS alone, ONSD alone and the combined model are shown in (Figure 1 - see PDF). The combined model had the highest AUC (0.765, 95% CI 0.690-0.840), compared to GCS alone (0.722, 95% CI 0.642-0.803) and ONSD alone (0.677, 95% CI 0.594-0.759). Although the combined model showed numerically better discrimination, DeLong's test comparing the combined model to GCS alone was not statistically significant (p=0.141). The Youden-derived optimal cut-off for ONSD alone to predict CSBI was 4.875 mm. At this threshold, sensitivity was 45.8%, specificity 83.8%, positive predictive value 44.9% and negative predictive value 84.3%. The addition of ONSD to GCS significantly improved model fit on likelihood ratio testing (χ^2^ = 5.04, p = 0.025). The combined model showed a lower AIC (195.8 vs. 198.9) and a higher Nagelkerke R^2^ (0.251 vs. 0.220), indicating better overall performance and increased explained variance. The categorical NRI for risk thresholds 0.3-0.6 was -0.066 (95% CI -0.119 to 0.220) and the IDI was 0.022 (95% CI 0.000 to 0.087). These reclassification indices showed a favourable trend, though they did not reach statistical significance. Bootstrap validation with 1000 resamples showed minimal optimism (0.011) and an optimism-corrected c-index of approximately 0.76, indicating good internal validity and low overfitting. The calibration plot (Figure 2 - see PDF) showed close agreement between predicted and observed probabilities, with a mean absolute error of 0.03, demonstrating excellent calibration. Decision curve analysis (Figure 3 - see PDF) demonstrated that the combined ONSD and GCS model provided greater net benefit than both the "treat all" and "treat none" strategies across threshold probabilities of approximately 0.1-0.6. Importantly, the combined model yielded higher net benefit than GCS alone in the clinically relevant threshold range of 0.3-0.6, supporting its added clinical utility for guiding decisions such as CT imaging or neurosurgical referral. ONSD increased progressively across higher Rotterdam scores and Marshall Grades (Figure 4 - see PDF). The Jonckheere-Terpstra test for trend was highly significant (p < 0.001), confirming a monotonic dose-response relationship between ONSD and CT-defined injury severity. In exploratory ROC analysis, ONSD showed fair discriminative ability for predicting a structurally severe Rotterdam score (≥3), with an AUC of 0.73 (95% CI: 0.586-0.730) (Figure 6 - see PDF). The optimal Youden cut-off was 5.1 mm, yielding a sensitivity of 50.0% and a specificity of 87.1%. However, after adjusting for GCS, the association between ONSD and Rotterdam score ≥3 was positive but not statistically significant (OR ≈ 2.0, 95% CI 0.90-4.37, p = 0.087). No independent association was found with Marshall Grade ≥III. GCS remained a consistent independent predictor of severe CT findings in both models. Given the small number of patients with Rotterdam score ≥3 (n=14), these results should be interpreted cautiously. Reinforcing the dose-response gradient, mean ONSD was highest in the GCS-defined severe TBI group (GCS 3-8: 5.67 ± 0.88 mm vs. 4.29 ± 0.68 mm in non-severe, p < 0.001). When treated descriptively, ONSD discriminated GCS-defined severe from non-severe TBI with an AUC of 0.885 (95% CI 0.776-0.994) and a cut-off of 5.4 mm (sensitivity 72.7%, specificity 92.6%) (Figure 6 - see PDF). This observation is descriptive only, as GCS it defines the groups and does not imply an independent predictive role for ONSD in these patients. To translate the combined model into a clinically useful tool, we derived a pragmatic four-tier approach (Table 3) based on the primary CSBI results (using a rounded ONSD threshold of 5.0 mm for simplicity).

## Discussion:

This prospective study of 215 head-injury patients demonstrates that a combined model incorporating optic nerve sheath diameter measured by bedside ultrasound and the Glasgow Coma Scale can predict clinically significant brain injury with fair-to-good discrimination (AUC = 0.765) and excellent calibration. ONSD remained an independent predictor of CSBI after adjusting for GCS and age (OR = 1.74 per 1 mm increase, 95% CI 1.04-2.92). More importantly, decision curve analysis showed that the combined model offers greater net clinical benefit than GCS alone over the clinically relevant threshold range of 0.3-0.6. Adding ONSD to GCS significantly improved model fit (likelihood ratio test χ^2^ = 5.04, p = 0.025) and explained variance and the strong dose-response relationship between ONSD and CT severity (Jonckheere-Terpstra p < 0.001) supports the biological plausibility of ONSD as a marker of intracranial pathology [6]. Our observation that ONSD is independently associated with a composite outcome of clinically significant brain injury aligns with previous studies. A systematic review and meta-analysis by Chen *et al.* (2023) including eight prospective studies with 222 TBI patients reported a pooled sensitivity of 0.82 and specificity of 0.82 for sonographic ONSD in detecting elevated ICP, with an AUC of 0.87 [7]. The lower AUC in our combined model (0.765) may be explained by our use of a composite CT-based outcome rather than invasive ICP measurement and by the inclusion of a substantial proportion of mild and moderate injuries. Other studies using CT findings as the reference standard have reported comparable AUCs in the range of 0.73-0.85 [8]. Several studies have examined the correlation between ONSD and GCS, consistently showing higher ONSD values in patients with lower GCS scores. For example, Chen *et al.* and Kshirsagar *et al.* reported mean ONSD values of 3.89 mm, 4.50 mm and 4.81 mm for mild, moderate and severe TBI, respectively [7, 9]. Our observed ONSD values in CSBI patients (median 4.8 mm) agree with these findings. Previous research has also established that ONSD >5.0 mm has good sensitivity and specificity for detecting abnormal CT findings. The optimal ONSD cut-off we derived for CSBI (4.875 mm) is close to this literature-derived threshold and the Youden cut-off for the exploratory Rotterdam ≥3 analysis (5.1 mm) further reinforces the consistency. The added value of combining ONSD with GCS has been investigated in a few studies, with some showing improved prediction of in-hospital mortality compared to GCS alone [10]. Our study extends this by using a more clinically relevant composite outcome (CSBI) and by quantifying added value through likelihood ratio tests, AIC, R^2^ and reclassification indices metrics not commonly applied in ONSD research. Additionally, we performed decision curve analysis, which demonstrated clear clinical utility in the 0.3-0.6 threshold range, even though the AUC improvement over GCS alone was not statistically significant (p = 0.141). This highlights a key lesson: statistical significance of AUC improvement is not the same as clinical utility. DCA directly quantifies whether a model leads to better decisions and is increasingly recommended for prediction model studies [11]. The monotonic dose-response relationship between ONSD and CT severity (Jonckheere-Terpstra p < 0.001) adds biological plausibility to our results. Similar trends have been reported in CT-based studies, where larger ONSD was associated with midline shift after severe TBI [12]. In an exploratory analysis, ONSD showed fair discriminative ability for predicting Rotterdam score ≥3 (AUC 0.73), but the association lost significance after adjusting for GCS, likely due to the small number of patients with this finding (n = 14). The absence of an independent association with Marshall Grade ≥III may reflect differences in what these scores capture: the Rotterdam score incorporates basal cistern effacement and midline shift (both closely tied to ICP), whereas the Marshall grade focuses more on mass effect and surgical lesion type [13]. ONSD may be more directly linked to ICP dynamics than to the presence of a surgical mass lesion. This mechanistic insight supports the use of ONSD as a physiological marker rather than a purely anatomical one. For completeness, we also examined ONSD's performance for the most severe radiological phenotype - Rotterdam score ≥4 (compressed basal cisterns, midline shift and SAH). In this exploratory analysis, ONSD discriminated remarkably well (AUC 0.935, optimal cut-off 5.2 mm), but the finding is limited by the very small number of patients (n = 1) and should be considered hypothesis-generating only, not a basis for clinical decision-making. To translate the combined model into a tool that can be used at the bedside, we derived a pragmatic four-tier risk stratification based on GCS and a rounded ONSD threshold of 5.0 mm (Table 3). This table uses only the primary CSBI results and incorporates the exploratory finding that an ONSD ≥5.4 mm or Rotterdam ≥3 denotes extreme risk. Such an approach can help emergency physicians triage patients in settings where CT is not immediately available (e.g., primary health centres, rural hospitals). For example, a patient with GCS 15 and ONSD <5.0 mm (low risk) may safely avoid transfer for CT, whereas a patient with GCS ≤13 or ONSD ≥5.0 mm (high risk) should undergo urgent imaging or be transferred. The very high-risk tier (GCS ≤8 or any two of ONSD ≥5.4 mm, Rotterdam ≥3, midline shift) signals the need for immediate CT and neurosurgical consultation. This algorithm, while requiring external validation, offers a simple and reproducible way to integrate ONSD into existing triage protocols.

## Strengths and limitations:

This study has several strengths. First, it uses a composite outcome (CSBI) that reflects both clinical and radiological severity, avoiding reliance on any single CT finding. Second, we performed rigorous internal validation using bootstrapping, calibration analysis and decision curve analysis methods often absent in ONSD prediction studies. Third, the prospective design and real-world emergency department setting in a lower-middle-income country enhance generalizability. Fourth, the dose-response assessment strengthens the mechanistic argument for ONSD as a marker of intracranial pathology. Fifth, we used a standardised ONSD measurement protocol (average of both eyes, 3 mm posterior to the globe) that is reproducible and validated. Limitations include the single-centre design and modest sample size (48 CSBI events), limiting statistical power (e.g., wide CI for ONSD OR 1.04-2.92). We lacked invasive ICP measurements. Exploratory Rotterdam ROC analyses were based on very small event numbers (n=14 for ≥3; n=1 for ≥4) and are hypothesis-generating only. External validation is needed. The optimal ONSD cut-off (4.875 mm) was rounded to 5.0 mm for the clinical algorithm as a pragmatic compromise.

## Conclusion:

We show the ONSD-GCS model provides fair discrimination, excellent calibration and greater net benefit than GCS alone. Adding ONSD improves model fit and the dose-response relationship supports biological validity. A four-tier bedside tool using GCS and an ONSD cut-off of 5.0 mm can aid triage, especially in resource-limited settings.

## Figures and Tables

**Table 1:** Baseline characteristics by CSBI status

**Variable**	**No CSBI (n=167)**	**CSBI (n=48)**	**p-value**
Age group, n (%)			0.258
<30 years	75 (44.9)	18 (37.5)	
30-60 years	77 (46.1)	28 (58.3)	
>60 years	15 (9.0)	2 (4.2)	
Gender, n (%)			0.904
Female	21 (12.6)	7 (14.6)	
Male	146 (87.4)	41 (85.4)	
GCS total, median (IQR)	15 (15-15)	13 (10-15)	<0.001
ONSD_avg, median (IQR)	4.1 (3.8-4.6)	4.8 (4.2-5.4)	<0.001

**Table 2:** Multivariable logistic regression for CSBI

**Variable**	**Adjusted OR**	**95% CI**	**p-value**
ONSD_avg (per 1 mm increase)	1.74	1.04-2.92	0.033
GCS.total (per 1 point increase)	0.73	0.62-0.85	<0.001
Age group (30-60 vs. <30)	1.08	0.51-2.31	0.842
Age group (>60 vs. <30)	0.4	0.05-1.80	0.289

**Table 3:** Proposed Bedside Risk Stratification Using GCS and ONSD for Predicting Clinically Significant Brain Injury

**Risk Tier**	**Criteria**	**Suggested Action**
Low	GCS 15 and ONSD <5.0 mm	Observation; CT may be deferred based on clinical judgement and decision rules
Intermediate	GCS 14 or ONSD 5.0-5.3 mm	Non-contrast CT head; monitored admission
High	GCS ≤13 or ONSD ≥5.0 mm (especially with GCS 14-15)	Urgent CT + neurosurgical consultation
Very High*	GCS ≤8 or any two of: ONSD ≥5.4 mm, Rotterdam ≥3, midline shift	Immediate CT and escalation to neurosurgical theatre
*Note: The "Very High" tier
incorporates the exploratory
finding that an ONSD ≥5.4 mm
or a Rotterdam ≥3 denotes extreme
risk. In settings without immediate CT,
patients in High/Very High tiers should
be transferred urgently.

## References

[1] Mayes KD (2026). Acad Emerg Med..

[2] https://www.ncbi.nlm.nih.gov/books/NBK513298/.

[3] Kriti K (2025). Cureus..

[4] Kaur A (2021). Ann Indian Acad Neurol..

[5] Serban NL (2026). Life (Basel)..

[6] Piovani D (2023). Healthcare (Basel)..

[7] Chen W (2023). Biomed Rep..

[8] Malhotra AK (2025). NPJ Digit Med..

[9] Kshirsagar SJ (2024). Acute Crit Care..

[10] https://www.ncbi.nlm.nih.gov/books/NBK554479/.

[11] Cook NR. (2018). Diagn Progn Res..

[12] Amakhian AO (2023). Cureus..

[13] Sharma PK (2024). Cureus..

